# Implant Rehabilitation Following Loss of Tooth Restorability Associated With Poorly Controlled Type 2 Diabetes Mellitus: A Case Report

**DOI:** 10.7759/cureus.113528

**Published:** 2026-07-28

**Authors:** Maira P Monroy Torres, Yelitza Rojas, Ana M Moreno Marin, Sidney Ramirez Galavis, Yaniet E Lorenzo Rivero

**Affiliations:** 1 Dentistry, Dra Pamela Monroy Clinica Odontologica y Estetica Integral IPS S.A.S., Villavicencio, COL; 2 Dentistry, Family Dental Home, Casselberry, USA; 3 Dentistry, Signature Dental Group, Delray Beach, USA; 4 Dentistry, Journey Dental, Kissimmee, USA; 5 Dentistry, Natural Smiles, Orlando, USA

**Keywords:** alveolar bone loss, delayed treatment, dental implant, implant rehabilitation, loss of restorability, periodontal disease, ridge preservation, tooth extraction, tooth mobility, type 2 diabetes mellitus

## Abstract

Type 2 diabetes mellitus is associated with an increased risk of periodontal disease, impaired bone metabolism, delayed wound healing, and progressive alveolar bone loss, all of which may adversely affect the prognosis of compromised teeth. This case report describes the management of a 42-year-old man with type 2 diabetes mellitus who presented with a severely compromised maxillary canine. The patient had previously been advised to undergo definitive restorative treatment with a full-coverage crown because of extensive structural deterioration. However, financial limitations, inconsistent medical follow-up, and repeated replacement of large restorations delayed definitive care. Over time, progressive structural deterioration, increasing tooth mobility, and loss of periodontal support rendered the tooth non-restorable. Initial medical evaluation revealed a glycated hemoglobin (HbA1c) level of 7.0%, indicating suboptimal glycemic control. The patient was referred to his primary care physician and counseled on improving glycemic control through adherence to prescribed metformin therapy, dietary modifications, and regular medical follow-up. After approximately one month of improved diabetes management, extraction of the tooth and ridge preservation using particulate bone graft and a resorbable collagen membrane were performed. Following six months of healing, a dental implant was placed, and after successful osseointegration, the definitive implant-supported crown was delivered. This case highlights the importance of timely definitive restorative treatment, optimization of glycemic control before implant therapy, and interdisciplinary collaboration in achieving predictable implant rehabilitation in patients with type 2 diabetes mellitus.

## Introduction

Type 2 diabetes mellitus is a chronic metabolic disorder that affects millions of individuals worldwide and is associated with numerous oral complications, including periodontal disease, impaired wound healing, increased susceptibility to infection, and progressive alveolar bone loss [1]. Persistent hyperglycemia adversely affects the host immune response and bone metabolism, increasing the risk of periodontal tissue destruction and compromising the long-term prognosis of natural teeth [2]. Appropriate glycemic control and regular medical follow-up are essential components of comprehensive dental care in patients with diabetes [3].

The preservation of structurally compromised teeth depends on timely diagnosis and definitive restorative intervention. Delaying appropriate treatment may allow continued structural deterioration, recurrent restorative failure, and progression of periodontal destruction, ultimately rendering a previously restorable tooth non-restorable. Financial limitations, inadequate patient compliance, and uncontrolled systemic conditions may further complicate treatment planning and adversely influence clinical outcomes [4].

Dental implant therapy has become a predictable treatment option for replacing non-restorable teeth; however, successful implant rehabilitation requires careful management of local and systemic risk factors [5]. Several clinical and systematic reviews have demonstrated favorable implant survival rates in patients with diabetes when adequate glycemic control is achieved [6-8]. Additional evidence supports the feasibility of implant therapy in diabetic patients, although careful patient selection and maintenance remain essential [9-11]. Furthermore, patient-reported outcomes indicate high levels of satisfaction and improved quality of life following implant rehabilitation in individuals with type 2 diabetes mellitus [12].

Diabetes-related alterations in wound healing and inflammatory responses may influence both hard and soft tissue healing around dental implants [13]. In addition, diabetes has been recognized as an important risk factor for periodontal disease, which may further affect long-term oral health and implant maintenance [14]. Recent systematic reviews continue to support the predictability of implant therapy in diabetic patients when appropriate systemic control is achieved [15].

Following successful osseointegration, prosthetic rehabilitation plays a critical role in restoring function and esthetics. Screw-retained implant crowns provide a predictable restorative option and facilitate retrievability and maintenance when compared with cement-retained alternatives [16]. Long-term implant success may also be influenced by biomechanical factors such as the crown-to-implant ratio, which has been evaluated in systematic reviews and clinical studies investigating implant survival, prosthetic complications, and marginal bone loss [17-19]. Recent long-term evidence has demonstrated favorable outcomes for single-implant-supported crowns restored with titanium base abutments, further supporting the predictability of implant-supported rehabilitation [20].

This case report describes the management of a 42-year-old man with type 2 diabetes mellitus who presented with a severely compromised maxillary canine following years of delayed definitive restorative treatment, repeated replacement of extensive restorations, and progressive structural and periodontal deterioration. The report highlights the importance of optimizing systemic health before implant therapy and demonstrates how appropriate treatment planning, ridge preservation, and delayed implant rehabilitation can successfully restore function and esthetics after the loss of a non-restorable tooth.

## Case presentation

A 42-year-old man with a history of type 2 diabetes mellitus presented for evaluation of a severely compromised maxillary left canine (tooth #11). The patient reported no known drug allergies and no other significant systemic medical conditions. He acknowledged inconsistent medical follow-up and poor adherence to dietary recommendations and antidiabetic therapy, resulting in suboptimal glycemic control. At presentation, his glycated hemoglobin (HbA1c) level was 7.0%.

The patient reported that several years earlier he had been advised by his general dentist to undergo definitive restorative treatment with a full-coverage crown because of extensive structural deterioration of tooth #11. However, because of financial limitations, he postponed the recommended treatment. Instead, he sought care at multiple dental offices over several years, where replacement of large restorations was repeatedly performed to alleviate symptoms. Although these restorations temporarily reduced discomfort, the patient continued to experience intermittent sensitivity to hot and cold stimuli and frequently self-medicated with over-the-counter analgesics, including ibuprofen, rather than pursuing definitive treatment.

**Figure 1:**
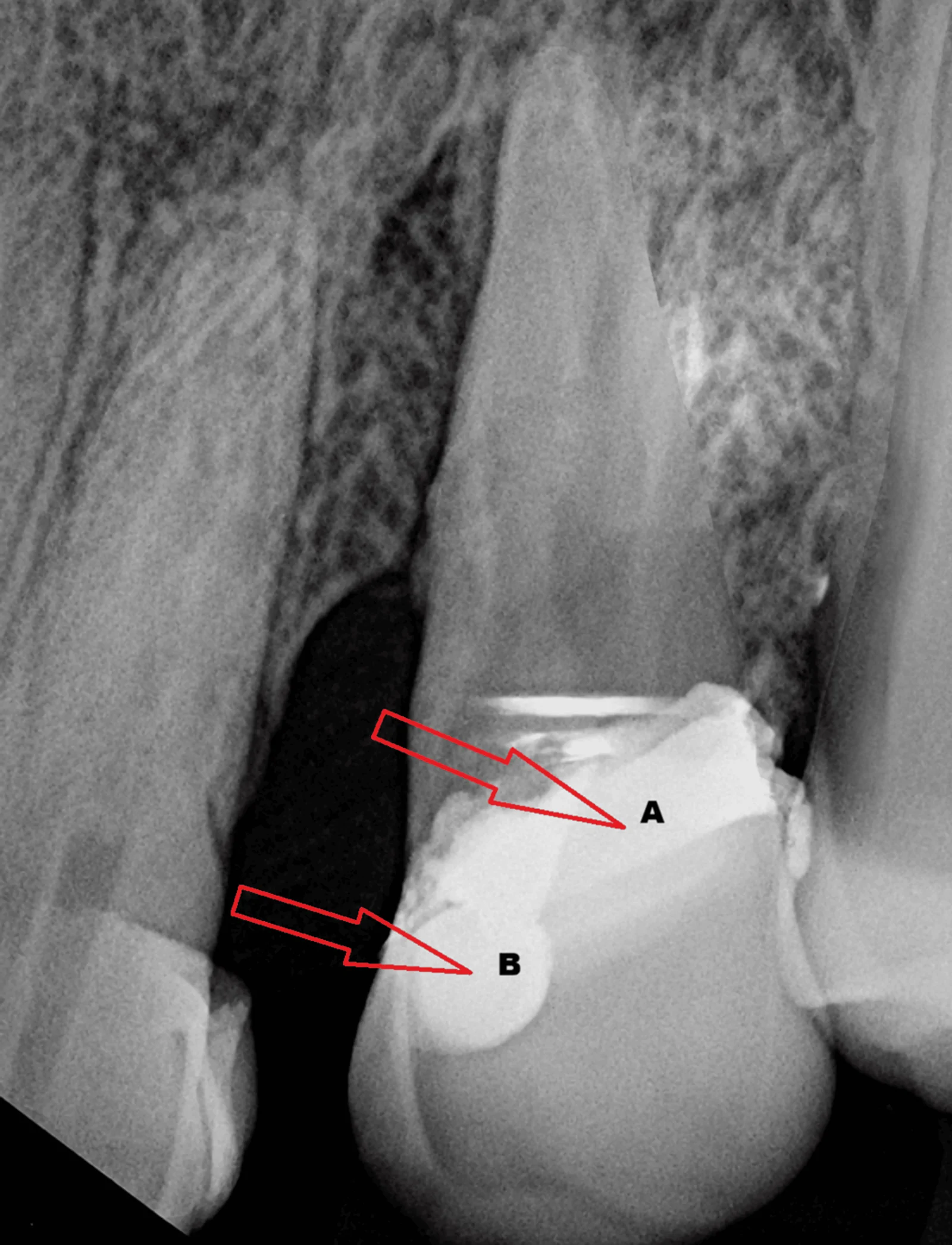
Preoperative Periapical Radiograph of Tooth #11. Preoperative radiograph demonstrating an extensive existing restoration (A) and limited remaining coronal tooth structure (B), resulting in an unfavorable restorative prognosis. Clinical examination also revealed increased tooth mobility associated with reduced periodontal support.

Over time, progressive structural deterioration of the remaining tooth structure, increasing tooth mobility, and loss of periodontal support resulted in an unfavorable restorative prognosis. Clinical examination revealed extensive coronal destruction with inadequate remaining tooth structure to support definitive restoration. The tooth demonstrated increased mobility, and radiographic examination showed extensive structural compromise with reduced supporting alveolar bone (Figure 1).

After comprehensive clinical and radiographic evaluation, the tooth was determined to be non-restorable. Treatment options, including extraction, ridge preservation, delayed implant placement, and implant-supported restoration, were discussed with the patient, who elected to proceed with this treatment plan.

Because of his suboptimal glycemic control, the patient was referred to his primary care physician before surgical treatment. Medical recommendations included improved adherence to prescribed metformin therapy, dietary modification, and regular medical follow-up. Approximately one month later, after demonstrating improved compliance with his medical management, dental treatment was initiated.

**Figure 2:**
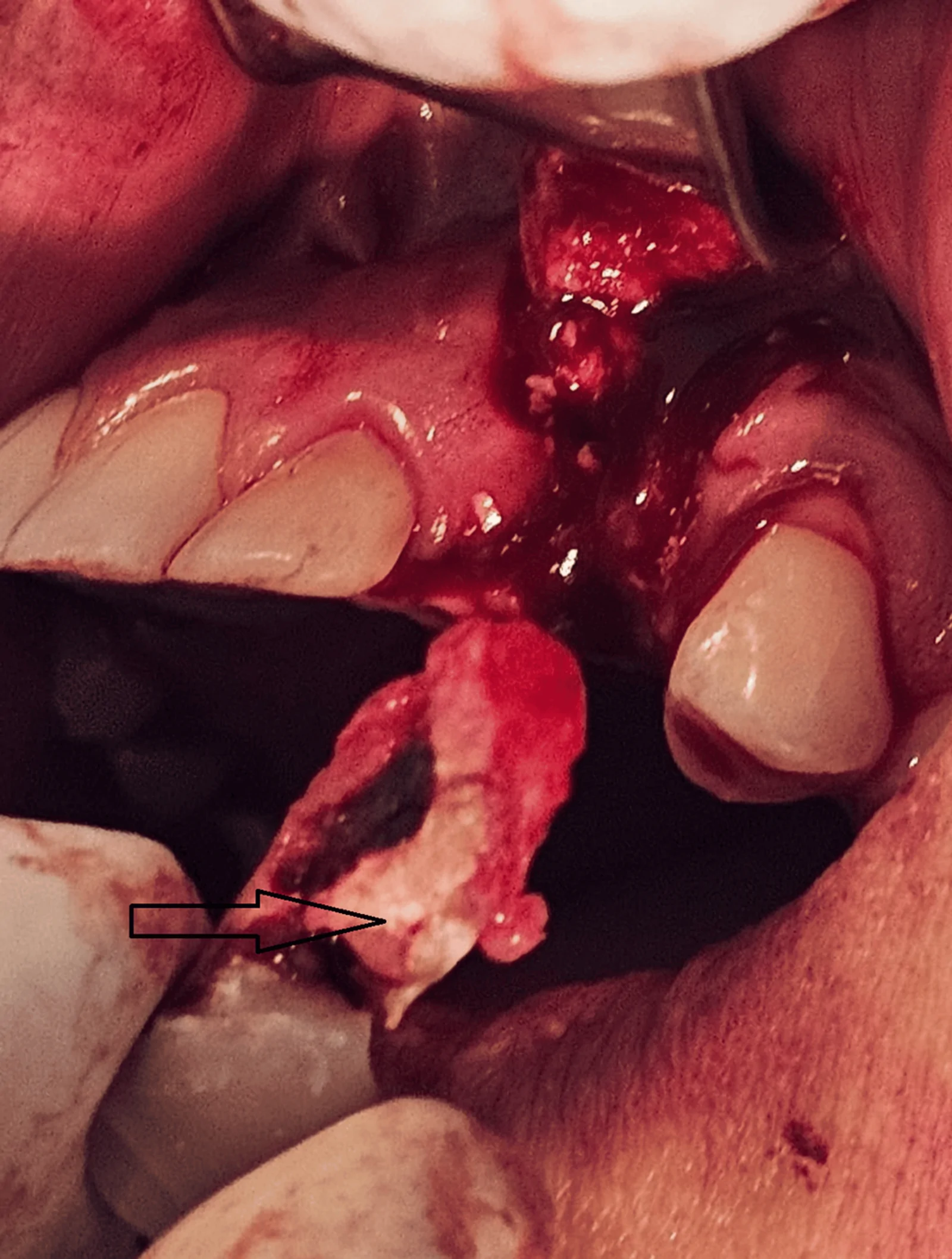
Clinical Photograph of the Extracted Tooth Following Surgical Extraction of Tooth #11. Intraoperative clinical photograph demonstrating the extracted maxillary canine. The black arrow indicates adherent inflammatory soft tissue attached to the root surface. Following extraction, the socket was thoroughly debrided to remove all inflammatory tissue before ridge preservation with particulate bone graft and a resorbable collagen membrane.

Following administration of local anesthesia, tooth #11 was carefully extracted. During the extraction procedure, the remaining alveolar bone was found to be thin and fragile, and partial fracture of the socket wall occurred during tooth removal because of the compromised supporting bone. The extracted tooth demonstrated adherent inflammatory soft tissue attached to the root surface (Figure 2).

**Figure 3:**
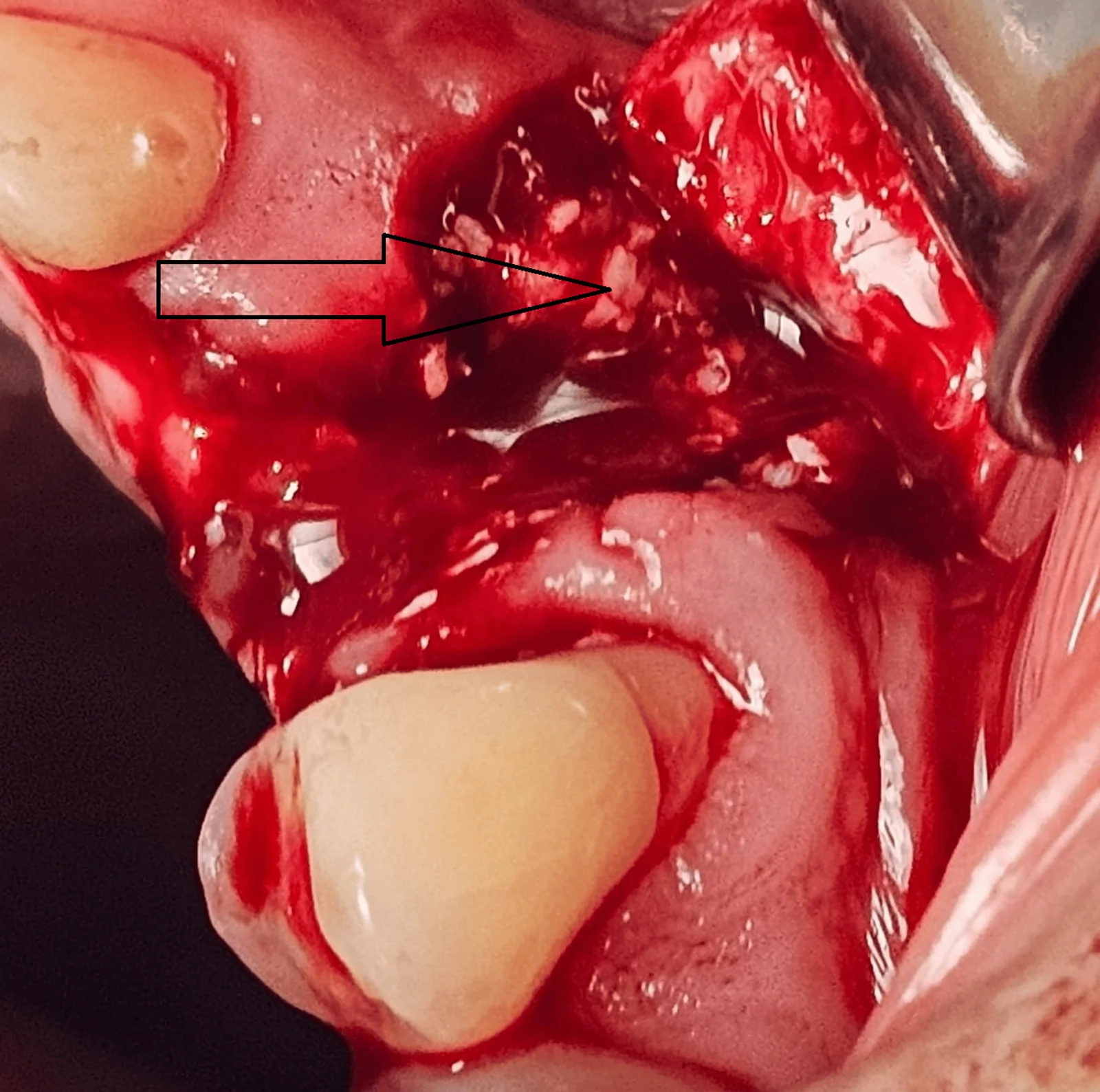
Placement of Particulate Bone Graft Following Extraction of Tooth #11. Intraoperative clinical photograph demonstrating placement of particulate bone graft within the extraction socket following complete debridement and irrigation. The black arrow indicates the particulate bone graft placed to preserve the alveolar ridge before coverage with a resorbable collagen membrane as part of the staged implant rehabilitation.

The extraction socket was thoroughly debrided, and all granulation tissue and non-viable soft tissue were removed. The surgical site was copiously irrigated with sterile saline before ridge preservation was performed using particulate bone graft material to preserve the alveolar ridge for future implant placement (Figure 3).

**Figure 4:**
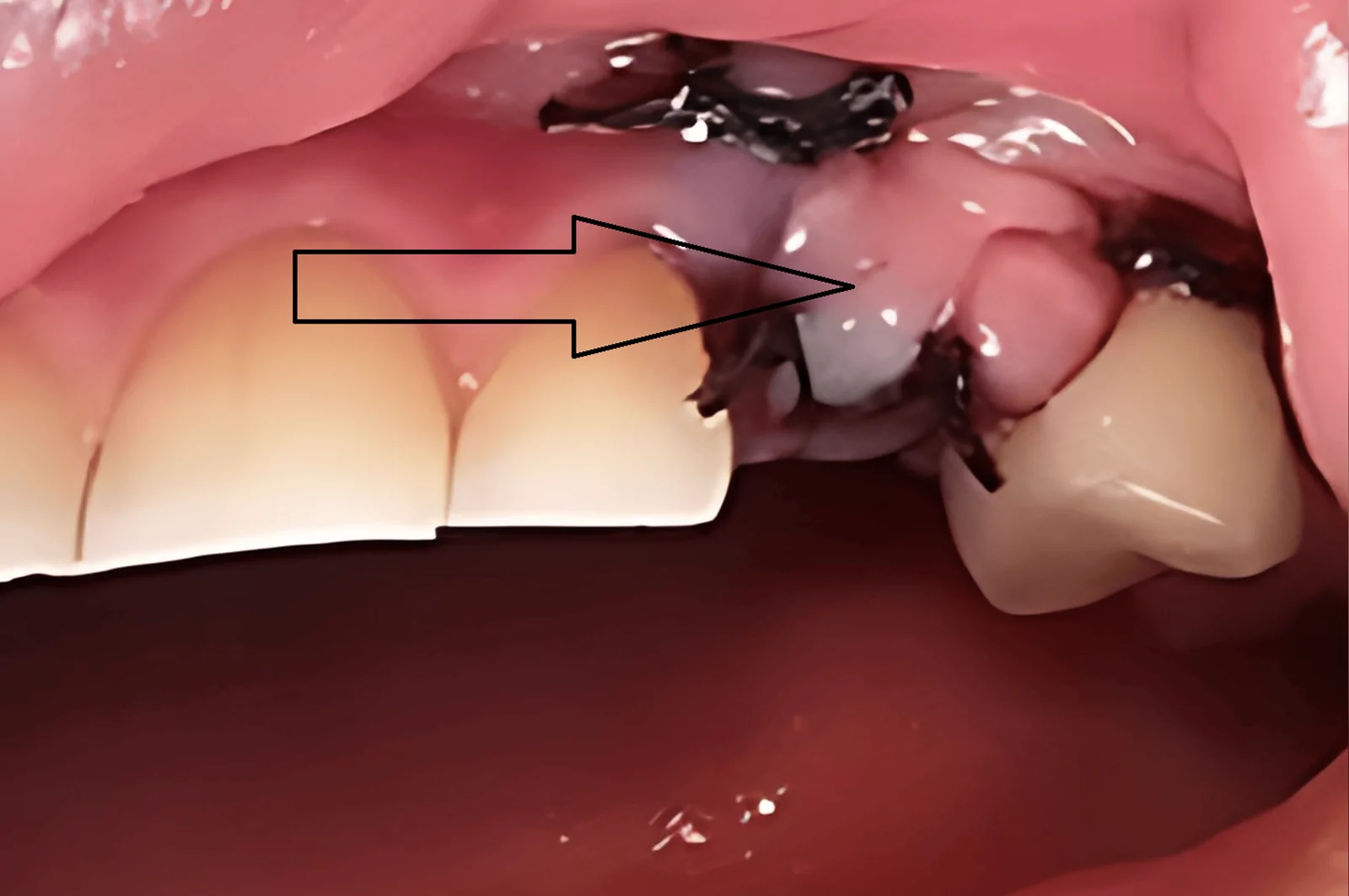
Placement of the Resorbable Collagen Membrane Following Ridge Preservation. Intraoperative clinical photograph demonstrating the resorbable collagen membrane (black arrow) positioned over the particulate bone graft to stabilize the graft material and support guided bone regeneration before primary wound closure.

A resorbable collagen membrane was then positioned over the graft to stabilize the graft material and promote guided bone regeneration before primary wound closure with 3-0 resorbable chromic catgut sutures (Figure 4).

Postoperative healing was uneventful. The resorbable sutures gradually degraded over approximately 13 to 18 days, and soft tissue healing progressed without complications. The patient remained compliant with postoperative instructions and continued medical follow-up for diabetes management.

**Figure 5:**
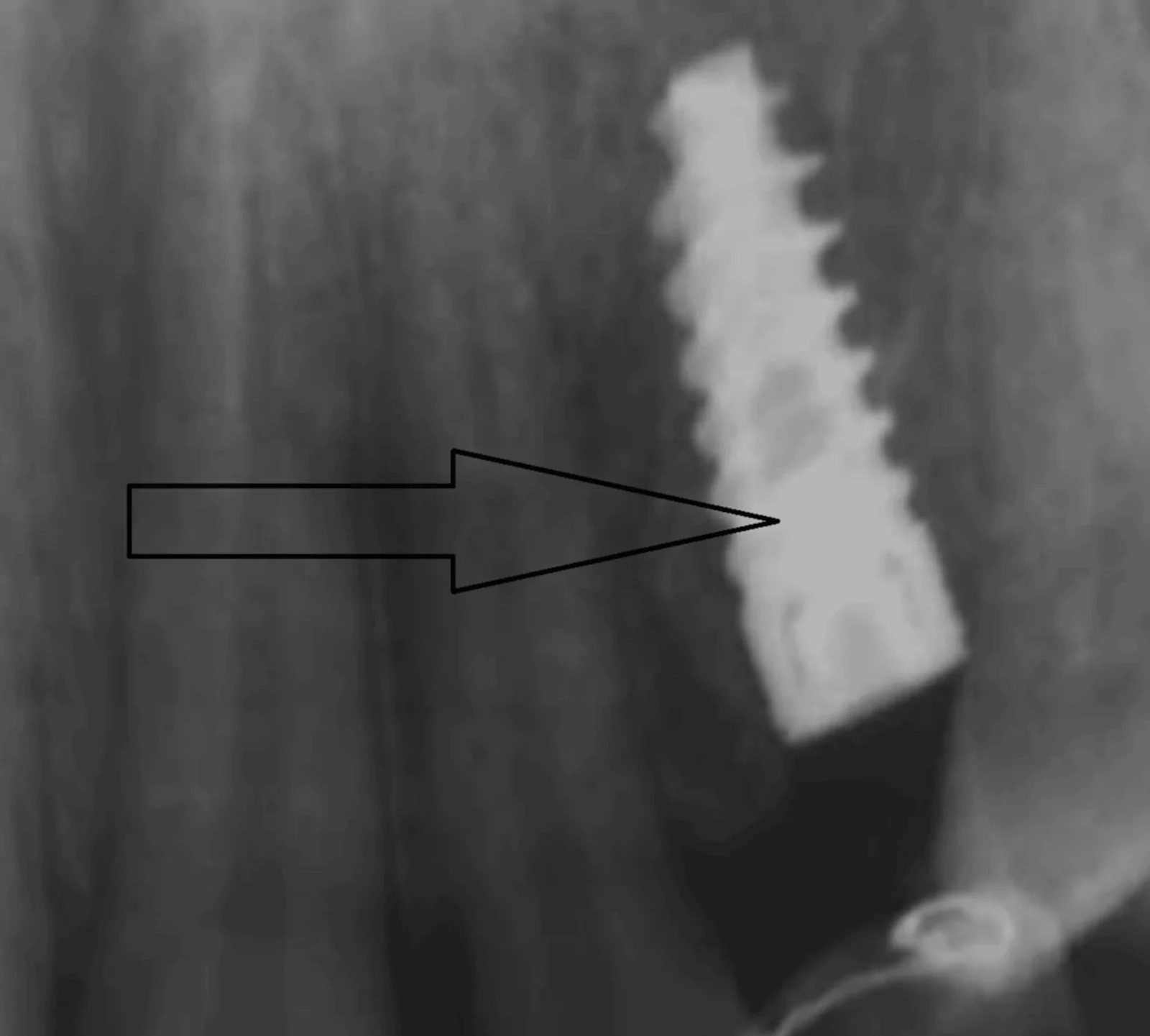
Postoperative Periapical Radiograph Following Implant Placement. Postoperative periapical radiograph demonstrating successful placement of the dental implant within the healed alveolar ridge after six months of ridge preservation. The black arrow indicates the implant positioned in the planned prosthetic location with satisfactory alignment and primary stability.

After six months of healing, clinical and radiographic evaluation demonstrated satisfactory preservation of the alveolar ridge with adequate bone volume for implant placement. A dental implant was placed following the manufacturer's recommended surgical protocol, achieving satisfactory primary stability (Figure 5).

**Figure 6:**
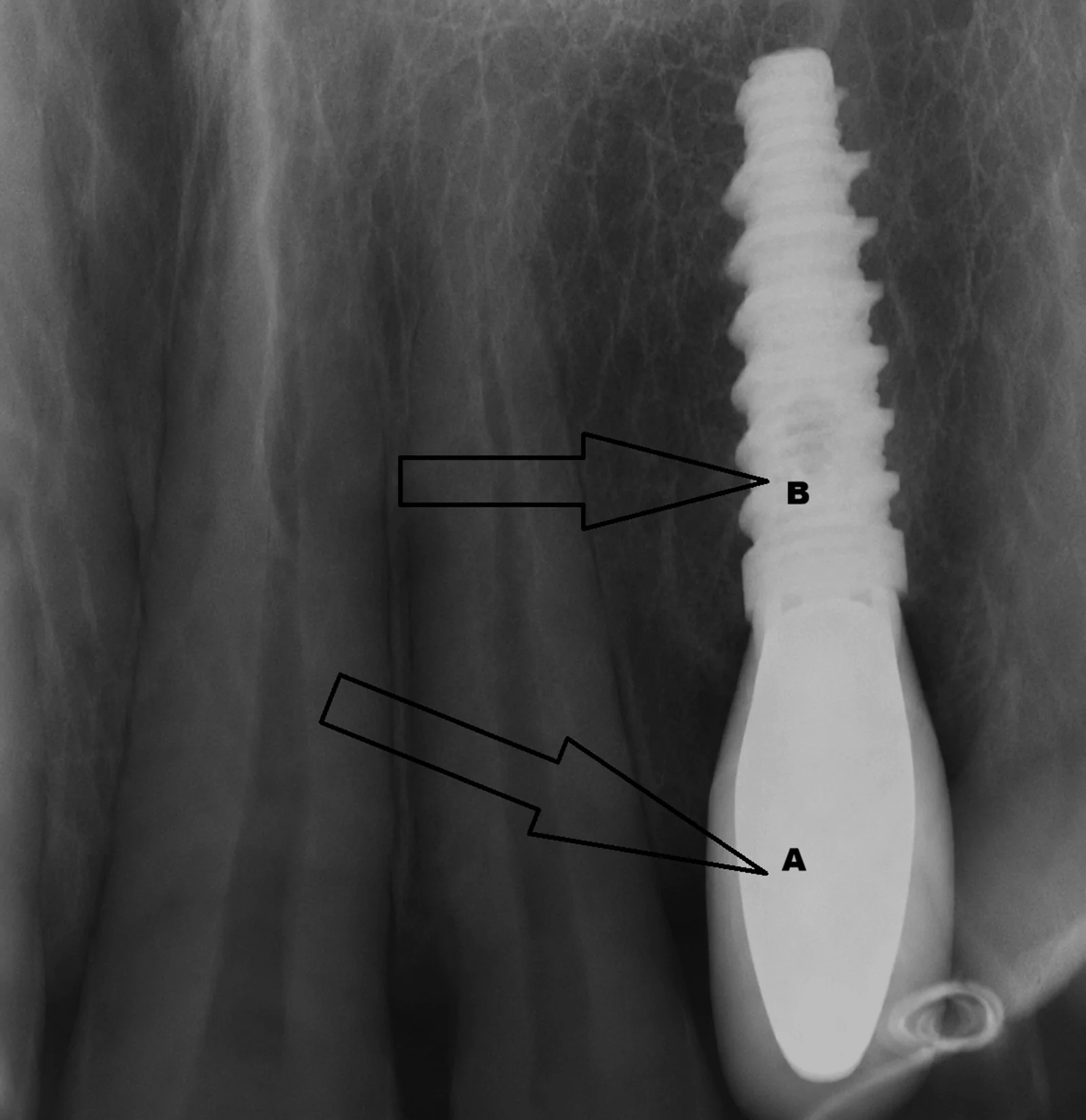
Definitive Implant-Supported Restoration Following Osseointegration. Postoperative periapical radiograph demonstrating the definitive implant-supported restoration after successful osseointegration. The lower black arrow (A) indicates the definitive implant-supported crown, while the upper black arrow (B) indicates the dental implant, demonstrating satisfactory positioning and favorable peri-implant bone support at the time of restoration delivery.

The implant was allowed to heal undisturbed for an additional three months to permit successful osseointegration. Following successful osseointegration, the restorative phase was completed with delivery of the definitive implant-supported crown. A postoperative periapical radiograph confirmed satisfactory seating of the definitive implant-supported crown (A) on the dental implant (B), demonstrating appropriate positioning and favorable peri-implant bone support at the time of restoration delivery (Figure 6).

Clinical evaluation demonstrated satisfactory esthetics, stable occlusion, healthy peri-implant soft tissues, and successful functional rehabilitation. The patient reported complete resolution of symptoms and expressed satisfaction with both the esthetic and functional outcome.

## Discussion

Poorly controlled type 2 diabetes mellitus may adversely affect dental implant therapy because hyperglycemia can compromise healing, osseointegration, implant stability, and peri-implant tissue health. Nevertheless, published reviews and clinical studies indicate that dental implants can achieve favorable survival outcomes in patients with diabetes when systemic health is carefully evaluated and glycemic control is appropriately managed [1-5].

Additional clinical evidence has demonstrated that diabetes may be associated with increased marginal bone loss and other biological complications, particularly when glycemic control is inadequate. However, successful implant rehabilitation has also been reported in diabetic patients when careful patient selection, appropriate surgical planning, and regular maintenance protocols are implemented [6-11]. Patient-reported evidence further suggests that implant treatment can improve satisfaction, oral function, and quality of life in individuals with type 2 diabetes mellitus [12].

Poor glycemic control is also associated with impaired healing of oral soft and hard tissues and an increased susceptibility to periodontal destruction. Persistent hyperglycemia promotes the formation of advanced glycation end products, oxidative stress, and the release of pro-inflammatory cytokines, which may alter the host immune response, impair bone metabolism, and compromise the supporting structures of natural teeth [13,14]. A recent systematic review further supports the general predictability of implant treatment in patients with diabetes while emphasizing the importance of glycemic control and appropriate clinical monitoring [15].

In the present case, delayed definitive restorative treatment, repeated replacement of extensive restorations, and suboptimal glycemic control likely acted together to produce progressive structural and periodontal deterioration. Although the tooth was initially considered restorable with definitive crown therapy, financial limitations resulted in postponement of treatment and repeated placement of large restorations rather than long-term rehabilitation. Over time, continued loss of tooth structure and deterioration of the supporting periodontal tissues rendered the tooth non-restorable, ultimately requiring extraction. This clinical progression emphasizes that delaying definitive treatment in structurally compromised teeth may substantially reduce the likelihood of preserving the natural dentition.

Successful implant therapy depends not only on appropriate surgical technique but also on careful management of systemic conditions that may influence healing. For this reason, the patient was referred to his primary care physician before implant placement to improve diabetes management through adherence to prescribed medication, dietary modification, and regular medical follow-up. This interdisciplinary approach reduced systemic risk factors before implant surgery and supported predictable healing throughout the rehabilitation process.

Ridge preservation following extraction was performed to minimize post-extraction alveolar ridge resorption and maintain sufficient bone volume for future implant placement. Preservation of the extraction socket was particularly important because maintenance of ridge dimensions facilitated prosthetically driven implant placement and reduced the potential need for more extensive augmentation. Following an appropriate healing period, adequate ridge preservation allowed successful implant placement and subsequent rehabilitation with a definitive screw-retained implant-supported crown [16].

Biomechanical and prosthetic factors must also be considered during implant restoration. Systematic reviews have indicated that an increased crown-to-implant ratio does not necessarily compromise implant survival; however, the ratio should be evaluated together with implant dimensions, occlusal loading, prosthetic design, marginal bone stability, and the distribution of functional forces [17-19]. Long-term prospective evidence has also demonstrated favorable clinical outcomes for single implant-supported crowns restored with titanium base abutments [20].

This case highlights the importance of patient education. Many patients postpone definitive treatment because symptoms become intermittent or are temporarily relieved by replacement restorations or over-the-counter analgesics. However, temporary symptom relief does not prevent continued structural or periodontal deterioration. Comprehensive patient education regarding the consequences of delaying treatment, together with clear communication concerning the effects of diabetes on oral health and healing, may improve treatment acceptance and long-term clinical outcomes.

Although limited by the inherent nature of a single case report, this report demonstrates the importance of comprehensive treatment planning, optimization of systemic health before implant surgery, and interdisciplinary collaboration between dental and medical providers. Early definitive intervention, appropriate medical management, ridge preservation, and staged implant rehabilitation may improve functional and restorative outcomes in patients with type 2 diabetes mellitus.

## Conclusions

This case demonstrates how delayed definitive restorative treatment, together with suboptimal glycemic control associated with type 2 diabetes mellitus, may contribute to progressive structural deterioration, periodontal breakdown, and eventual loss of restorability of a compromised tooth. Comprehensive treatment planning, optimization of systemic health before implant surgery, ridge preservation following extraction, and a staged approach to implant rehabilitation resulted in a favorable functional and esthetic outcome. This report highlights the importance of early definitive intervention, interdisciplinary collaboration between dental and medical providers, and patient education regarding the impact of systemic disease and treatment delays on long-term oral health.
